# Normothermic Regional Perfusion Experience of Organ Procurement Organizations in the US

**DOI:** 10.1001/jamanetworkopen.2024.40130

**Published:** 2024-10-24

**Authors:** Marty T. Sellers, Jennifer L. Philip, Aleah L. Brubaker, Roxane L. Cauwels, Kristopher P. Croome, Jordan R. Hoffman, Nikole A. Neidlinger, Andrea M. Reynolds, Anji E. Wall, John M. Edwards

**Affiliations:** 1 Gift of Life Donor Program, Philadelphia, Pennsylvania; 2 University of Wisconsin Organ and Tissue Donation, Division of Transplantation, Department of Surgery, University of Wisconsin School of Medicine and Public Health, Madison; 3 Department of Surgery, Division of Transplantation and Hepatobiliary Surgery, University of California San Diego, La Jolla; 4 CONCORD: Consortium for Donation after Circulatory Death and Normothermic Regional Perfusion Outcomes Research and Development, La Jolla, California; 5 Association of Organ Procurement Organizations, McLean, Virginia; 6 Department of Transplant, Mayo Clinic Florida, Jacksonville; 7 University of Colorado Hospital, Anschutz Medical Campus, Division of Cardiothoracic Surgery, Aurora; 8 Annette C. and Harold C. Simmons Transplant Institute, Baylor University Medical Center, Dallas, Texas

## Abstract

**Importance:**

Despite the unmet need for donor organs, organ use from donation after circulatory determination of death (DCD) donors has been limited by inferior transplant outcomes. Normothermic regional perfusion (NRP) improves recipient outcomes and organ utilization from DCD donors. There is variability in NRP policies and experience among US organ procurement organizations (OPOs).

**Objectives:**

To determine OPO experience, identify operational inconsistencies, and explore needs related to NRP.

**Design, Setting, and Participants:**

This survey study included 55 OPOs in the US that had recovered DCD organs and completed a survey on operational, administrative, and educational components related to NRP in November to December 2023. Data analysis was performed from February to April 2024.

**Main Outcome and Measures:**

The primary outcome was the number of OPOs participating in and/or anticipating NRP participation. Secondary outcomes were NRP implementation barriers, OPO education practices, and future needs regarding consensus NRP recommendations and standards.

**Results:**

Of 55 respondents, 11 (20%) were chief executive officers, 8 (15%) were chief operating officers, and 36 (65%) were medical directors or chief clinical officers. Forty-nine OPOs facilitated NRP cases: 26 OPOs (53%) facilitated both thoracoabdominal NRP (TA-NRP) and abdominal NRP (A-NRP) cases, 16 OPOs (33%) facilitated only TA-NRP, and 7 OPOs (14%) facilitated only A-NRP. OPOs reported 606 NRP cases (421 TA-NRP [69%], 185 A-NRP [31%]); median (range) case experience was 8 (1-52). Fifty-two of 55 OPOs (95%) thought standardized guidance documents would be helpful. All 49 OPOs facilitated NRP at a transplant center’s request; 39 (80%) had NRP initiated by a nonlocal transplant center. Twenty-three of 49 OPOs (47%) participated in NRP without a policy and without a policy pending approval. Positive donor hospital feedback was received by 29 OPOs (59%), primarily focused on increased organs transplanted and prerecovery communication. Allocation challenges were experienced by 21 OPOs (43%); their median (range) case volume was higher than those with no reported allocation challenges (11 [3-52] vs 6.5 [1-29]; *P* = .03). Eleven OPOs (22%) had incorporated NRP into general donor hospital education.

**Conclusions:**

In this survey study of US OPOs, wide variation existed with respect to NRP experience and practice. Allocation challenges occurred more frequently with increased NRP experience. NRP guidelines and standardization were desired by most OPOs to decrease allocation challenges and maximize the gift of organ donation.

## Key Points

**Question:**

What are the experiences, practices, and perspectives of normothermic regional perfusion (NRP) by organ procurement organizations (OPOs) in the United States as of the end of 2023?

**Findings:**

In this survey study of 55 OPOs with 100% response rate, 49 OPOs reported experience with NRP. There was wide variation in NRP experience and practice; 52 OPOs desired standardized guidelines related to NRP.

**Meaning:**

These results suggest that improved standardization of NRP policies, protocols, and practices are desired and necessary to maximize organ donation and transplantation potential from donation after circulatory determination of death donors.

## Teaser

This survey study investigates organ procurement organizations’ experience, operational practices, and needs related to normothermic regional perfusion (NRP).

## Introduction

The number of donation after circulatory determination of death (DCD) donors in the US has substantially increased over the last 25 years.^1^ Over 25% of deceased donor transplants were with DCD organs in 2023, yet DCD organ use in the US lags substantially behind that in Europe.^1,2^ This unrealized potential is driven by historically inferior clinical outcomes related to the warm ischemic injury inherent to the DCD process after withdrawal of life-sustaining therapy (WLST) through the “hands-off” or “no-touch” period.^3,4,5^ Normothermic regional perfusion (NRP) provides in-situ oxygenated, whole blood perfusion to either the thoracic and abdominal cavity (thoracoabdominal NRP [TA-NRP]) or the abdominal cavity alone (abdominal NRP [A-NRP]) after declaration of death in a DCD donor. TA-NRP and A-NRP rehabilitates the ischemic injury incurred during the dying process, results in superior outcomes after kidney and liver transplantation, and increases utilization of organs compared with super rapid recovery (SRR) and static cold storage.^6,7,8,9,10,11,12,13,14,15^ The clinical benefits of NRP are well documented, spurring increased use of NRP in the US since 2020.^2,6,7,8,9,10,11,12,13,14,15,16,17^ However, a lack of consensus regarding NRP’s role in the national donation and transplantation landscape exists, resulting in nonuniform adoption and policy development among transplant centers and organ procurement organizations (OPOs).^18,19,20,21^ Process and implementation standardization is known to be helpful; and lack of standardization can result in patient outcome and quality of care differences.^22,23^ This emphasizes the need for consensus guidelines for any emerging recovery and/or perfusion technology, including NRP.

As NRP expands in the US, better understanding of the operational impact on OPOs is crucial, including relations and communication with donor hospitals and transplant centers, policy development, and consensus recommendations. Limited data have been generated to determine the association of NRP with OPOs and their integration of NRP into routine practice. Through a national survey to all 55 US OPOs performing DCD donation, information was collected to describe the end-of-2023 NRP status and identify needs surrounding NRP adoption, with the goal to determine areas where improved uniformity would facilitate increased organ availability and ultimately more life-saving transplants.^2,6,16^

## Methods

From November 7, 2023, through December 31, 2023, the Association for Organ Procurement Organizations (AOPO) conducted a voluntary online survey of the US OPOs to assess their experience with NRP. A team of clinicians, OPO staff, and AOPO leadership with expertise in organ donation and transplantation developed the questionnaire consisting of binary, multiple-choice, and free-response questions (eTable in Supplement 1). The 46-question survey assessed the OPOs’ entire experience with NRP (regardless of when their first case occurred), barriers to NRP implementation, hospital outreach and feedback, NRP education, and challenges and opportunities related to NRP. The survey was sent to 55 of 56 OPOs; the remaining OPO was excluded as they had not participated in DCD recoveries. There were no financial incentives for OPOs to participate. This survey study follows the American Association for Public Opinion Research (AAPOR) reporting guideline.^24^ The University of California San Diego institutional review board determined that this study was exempt from review and the requirement for informed consent because it represented minimal to no risk to the respondents who had voluntarily provided their OPO’s data.

Responses were collected using a secure web-based platform. OPOs were allowed 1 response from the chief executive officer (CEO), chief operating officer (COO), or medical director or chief clinical officer (CCO). Participants’ anonymity and confidentiality were ensured. Survey completion took approximately 15 minutes.

The primary outcome was to assess the number of OPOs that were actively participating in NRP programs or anticipating future NRP implementation. Secondary outcomes were identification of barriers to NRP implementation, OPO education practice patterns (internal staff, donor hospital, and family education patterns), and future needs regarding consensus NRP recommendations and standards. For ease of reporting, OPOs were categorized by the existing 11 Organ Procurement and Transplantation Network (OPTN) Regions.

### Statistical Analysis

Descriptive analysis of the data was used to identify patterns, trends, and associations among variables. Analyses were performed using Stata software 18.0 (Stata Corp) from February to April 2024. A *t* test or Mann-Whitney *U* test, as appropriate, was used to compare continuous variables, and Fisher exact test was used to compare proportions. A 2-sided *P* ≤ .05 was considered significant.

## Results

**Figure 1.**
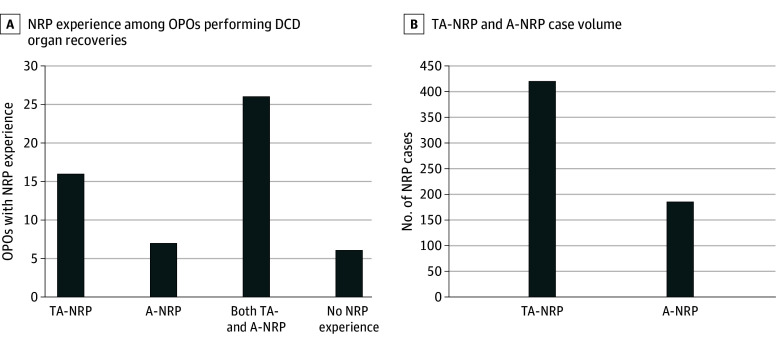
Normothermic Regional Perfusion (NRP) Experience and Case Volume Among Organ Procurement Organizations (OPOs) NRP experience among 55 OPOs (A) and case volume among 49 OPOs with NRP experience (B). A-NRP indicates abdominal NRP; DCD, donation after circulatory determination of death; TA-NRP, thoracoabdominal NRP.

**Figure 2.**
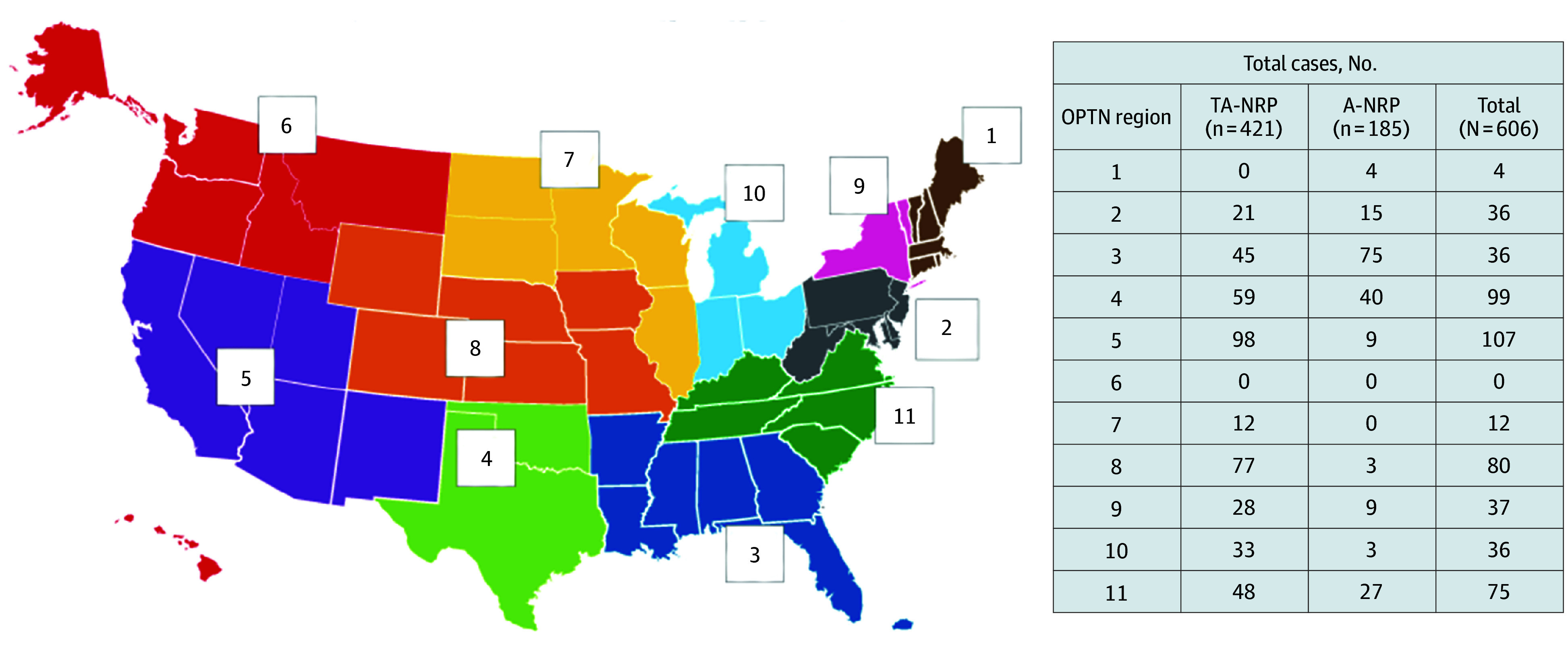
Normothermic Regional Perfusion (NRP) Case Volume by Organ Procurement and Transplantation Network (OPTN) Region A-NRP indicates abdominal NRP; TA-NRP, thoracoabdominal NRP.

Of 55 respondents (100% response rate), 11 (20%) were CEOs, 8 (15%) were COOs, and 36 (65%) were medical directors or CCOs. Forty-nine (89%) OPOs reported NRP experience: 26 (53%) both TA-NRP and A -NRP, 16 (33%) only TA-NRP, and 7 (14%) only A-NRP cases. Six OPOs (11%) had not facilitated NRP (Figure 1A). All OPOs had facilitated NRP at the request of a transplant center; 39 of 49 (80%) had NRP cases initiated by a transplant center outside their donation service area, and 23 (47%) had facilitated NRP for a transplant center within their donation service area. In total, 49 OPOs had facilitated 606 NRP cases (421 TA-NRP [69%]; 185 A-NRP [31%]) (Figure 1B). Total cases by OPTN region are shown in Figure 2.

**Figure 3.**
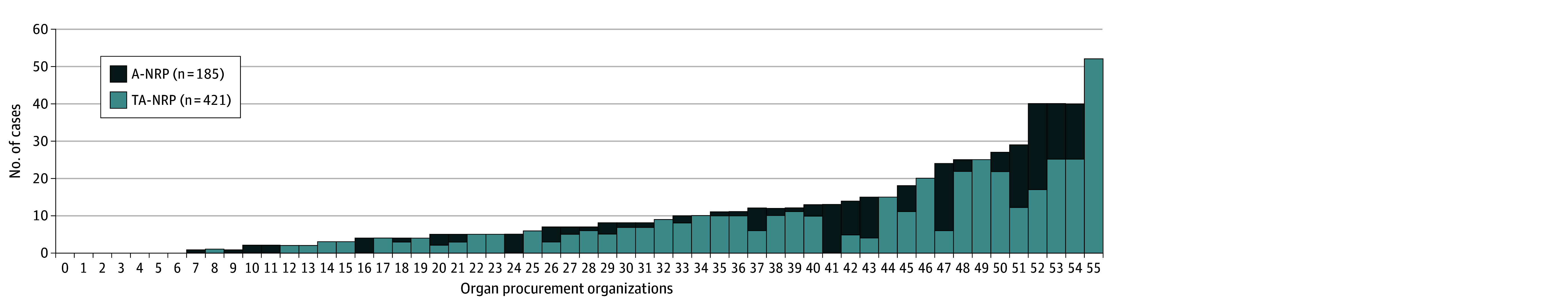
Thoracoabdominal Normothermic Regional Perfusion (TA-NRP) and Abdominal NRP (A-NRP) Case Volume per Organ Procurement Organization (Deidentified)

The median (range) number of NRP cases was 8 (1-52) and varied by experience with NRP case type. In the 26 OPOs with both TA-NRP and A-NRP experience, the median (range) number of total cases was 12 (1-40); the median (range) number of TA-NRP and A-NRP cases was 7 (2-25) and 3 (1-23), respectively. For the 16 OPOs with only TA-NRP experience, the median (range) number of cases was 5 (1-52), with only 1 OPO reporting more than 25 cases. In the 7 OPOs with only A-NRP experience, the median (range) number of cases was 2 (1-13), with only 1 OPO reporting more than 5 cases (Figure 3).

### Desire for Guidance and/or Standardization

Fifty-two of 55 OPOs (95%), including all 6 OPOs who had not participated in NRP, reported that standardized training materials and/or resources to develop and/or refine their NRP process would be helpful. Aggregate responses are shown in Table 1.

**Table 1.** Potential Resources and OPOs Indicating a Particular Resource Would be Beneficial

Potential resource	OPOs indicating the resource would be helpful, No. (%)
Training material for donor hospital personnel	45 (82)
Workflow checklist and resource guide	44 (80)
Most effective practices guidelines	42 (76)
Training materials for OPO staff	42 (76)
NRP language samples	38 (69)
General education for nonclinical staff/social workers	29 (53)
Training and/or technical details for your OPO recovery surgeons	27 (49)
Consent documents for NRP	26 (47)
Guide to navigating C-suite^a^ engagement	26 (47)
Training and/or technical details for local transplant center surgeon(s)	25 (45)

Abbreviations: NRP, normothermic regional perfusion; OPO, organ procurement organizations.

^a^ The highest-ranking executives in an organization (ie, chief-level).

### OPO Policies, Barriers, and Partner Hospital Processes

An approved NRP-specific policy was reported by 21 OPOs (43%) with NRP experience; an additional 5 (10%) had a policy pending approval. Twenty-three OPOs (47%) facilitated NRP cases in the absence of an approved policy.

No barrier with partner hospitals to perform NRP were reported by 31 OPOs (63%); 18 (37%) reported that at least 1 partner hospital declined NRP participation, with a median (range) of 3 (1-19) hospitals disallowing an NRP recovery, representing up to 25% of hospitals in their donation service area. The median number of total NRP cases in OPOs reporting a barrier with partner hospitals was 13, compared with 10 for those reporting no barrier (*P* = .26). Cited reasons for NRP nonparticipation included ethical concerns (16 of 18 [89%]), the American College of Physicians (ACP) Statement (4 of 18 [22%]), and religious concerns (4 of 18 [22%]).^18^ Two of 49 OPOs (4%) reported having at least 1 hospital in their donation service area that allowed A-NRP but prohibited TA-NRP for reasons that included ethical concerns, the ACP Statement, and concerns about resources and family disclosures.^18^

The 6 OPOs (11%) reporting no NRP experience cited legal concerns (n = 2), ethical concerns (n = 1), the ACP Statement (n = 1), and concerns about negative public relations or media (n = 1) as barriers, with 1 OPO not providing information.^18^ Five of these 6 OPOs (83%) planned to perform NRP in 2024.

Hospital notification was variable: 36 OPOs (73%) with NRP experience had notified the hospital that NRP would occur, and 13 (27%) had not, citing NRP as a postmortem perfusion technique, akin to other perfusion techniques not requiring prenotification. Of the 36 OPOs that notify hospitals about NRP, 26 (72%) notified the operating room and 16 (44%) notified critical care personnel. In addition to hospital notification, 11 of 36 OPOs (31%) requested permission to perform NRP, 4 of whom had spoken with hospital ethics committees or legal teams.

Thirty-nine of 49 OPOs (80%) who had performed NRP and all 6 who had not performed NRP stated the need for standardized hospital training materials. Real-time education to involve hospital personnel on an NRP case–specific basis was provided by 31 of 49 OPOs (63%); 11 (22%) incorporated NRP information into their general donation education, and 7 (14%) had targeted specific hospitals for NRP education. Despite standard, real-time NRP education across the majority of OPOs, most OPOs (26 of 31 [84%]) had not developed specific training materials.

Most OPOs with NRP experience (29 of 49 [59%]) received positive feedback from donor hospitals on the NRP process. Two primary reasons emerged from free-text responses: (1) increased number of organs transplanted and/or lives saved and (2) increased prerecovery communication. The median (range) number of cases where OPOs received positive feedback (10 [1-52]) was not significantly different for OPOs who had not received positive feedback (7.5 [1-29]; *P* = .55). For OPOs that reported a negative response to the NRP process by donor hospitals, 11 OPOs (22%) cited concerns by hospital administration, 7 OPOs (14%) reported concerns by critical care personnel, and 13 OPOs (27%) cited concerns by operating room personnel. More granular detail on these negative reactions were not provided. Additional concerns from free-text responses included the requirement for packed red blood cell availability and need for intraoperative point-of-care testing (1 OPO each). At least 1 technical failure during the recovery was reported by 15 OPOs (31%); the number of failures was not queried; those reporting at least 1 technical failure facilitated a median (range) of 13 (2-52) cases compared with 7 (1-40) for those without a technical failure (*P* = .03).

### Process and Technical Considerations for DCD Donors and Associations With NRP

Transfer of potential DCD donors to a dedicated hospital for recovery was reported by 8 of 55 OPOs (15%); 5 (9%) facilitate NRP in their dedicated hospital, and 3 (5%) OPOs transfer for the sole purpose of NRP. Fifty-two of 55 OPOs (95%) complete WLST for a potential DCD donor in the operating room. Eleven of 55 OPOs (20%) that perform DCD reported not allowing prepping and draping prior to WLST for a non-NRP donor; 10 (18%) allow prepping and draping during the 5-minute hands-off period and 3 (5%) of these allow prepping and draping prior to WLST if NRP is planned. One OPO does not prep and drape any DCD donor, regardless of NRP, until after the observation period.

### Prerecovery Communication

Most OPOs (47 of 49 [96%]) perform routine prerecovery huddles after all teams arrive on-site for NRP recovery. Conducting a prearrival huddle to facilitate discussion about logistical concerns and to minimize communication gaps was reported by 38 of 49 OPOs (78%). Table 2 shows specific agenda items in this huddle and the variability to which these items were included. Twenty-one of 49 (43%) reported poor communication with the transplant center requesting and performing NRP. The median (range) number of cases for those reporting poor communication was 12 (1-52) compared with 6 (1-25) cases for those not reporting poor communication (*P* = .03). There was no significant association between those reporting poor communication and those who conduct a prearrival huddle.

**Table 2.** Discussion Items Included in Prearrival Huddle Prior to an NRP Case^a^

Discussion item	OPOs, No. (%) (N=38)
Confirm type of NRP, TA-NRP vs A-NRP	36 (95)
Review 5-min hands-off period after death determined	34 (89)
Review transplant team communication expectations while participating in the recovery	34 (89)
Review the location of withdrawal of support (OR vs other)	34 (89)
Review the need for prerecovery huddle at donor hospital	33 (87)
Number of PRBCs the transplant team needs in the OR from the outset of case	33 (87)
Determine each transplant team’s threshold for FWIT and associated cutoff times	29 (76)
Will the patient require reintubation, and if so, what is the plan for reintubation?	29 (76)
Review head vessels will be clamped in TA-NRP cases at outset of the case	27 (71)
Determine if NRP will be pursued if the patient does not die within the specified timeframe for FWIT	27 (71)
Review transportation needs (air/ground) for transplant teams	25 (66)
Communicate the plan for prepping and draping the patient and the timing approved by the donor family	24 (63)
Length of time each team wants to remain on circuit (often heart teams would prefer shorter times compared to liver teams)	24 (63)
Will perfusion have additional needs (ie, oxygen tanks and vacuum lines)?	23 (61)
Required laboratory tests and who will bring the POC device to obtain laboratory test results	22 (58)
Will the heart team need a TEE, and if so, what is the plan for facilitating?	22 (58)
If more than 1 team interested in NRP, determine who will bring the ECMO equipment/personnel	20 (53)
Will the lung team need a bronchoscopy, and if so, what is the plan for facilitating?	20 (53)
Will transplant teams bring their own medications (vasopressors/inotrope)?	17 (45)
Will the patient come off circuit and stay off circuit after heart evaluation? Or will the patient go back on circuit?	17 (45)
Consider if there are laboratory test requirements of the donor hospital that cannot be performed by transplant teams with POC device	14 (37)
Other	6 (16)

Abbreviations: A-NRP, abdominal NRP; ECMO, extracorporeal membrane oxygenation; FWIT, functional warm ischemic time; NRP, normothermic regional perfusion; OR, operating room; POC, point of care; TA-NRP, thoracoabdominal NRP; TEE, transesophageal echocardiogram.

^a^ Prearrival huddles were conducted by 38 OPOs.

### Allocation Challenges and NRP

Experiences with allocation challenges on NRP cases were reported by 22 OPOs (45%); 13 (27%) indicated that the these challenges were intermittent and easily addressed; 7 (14%) indicated that the challenges happen routinely and need to be addressed immediately; and 1 (2%) reported challenges but proceed with NRP as standard of care and do not defer to transplant centers who do not want NRP; 1 (2%) reported challenges, but they were experienced rarely and free-texted a “few instances” where a lung recovery team did not want A-NRP to be performed. Conflict resolution included holding a conference call between accepting centers (15 of 49 [31%]) and autonomous decision-making by OPO medical and/or administrative leadership (6 of 49 [12%]). Those reporting allocation challenges had facilitated a median (range) of 11 (3-52) cases, compared with 6.5 (1-29) cases for those who had not experienced these challenges (*P* = .03).

Having coordinated at least 1 NRP recovery when another recovery and/or perfusion modality was used was reported by 33 of 49 OPOs (67%); 16 (33%) did not have experience combining NRP with another recovery/perfusion modality. Examples cited included: TA-NRP or A-NRP and normothermic machine perfusion (NMP) of the liver (n = 28), A-NRP and SRR with or without NMP of thoracic organs (n = 20), and a combination of these additional modalities (n = 13). Allocation challenges were reported by 18 of 33 OPOs (55%) who had combined NRP with other modalities vs 4 of 16 (25%) who had not (*P* = .07).

## Discussion

Our findings that virtually all OPOs expressed a desire for information and/or resources to develop and/or refine their NRP process should prompt action. Given the expansion of NRP across the US and our finding that the majority of OPOs have participated in NRP recoveries, it seems clear that this recovery technique will continue to be used as part of DCD donation. Recent reports show increased organ utilization from DCD donors with NRP compared with SRR in the US.^6,16^ Coupled with improved posttransplant outcomes seen with advanced perfusion techniques, including NRP, SRR with static cold storage of DCD organs may no longer be considered an acceptable standard of care.^25^ Our findings support a priority to develop standardized NRP processes, practices, procedures, policies, and training for OPOs, donor hospitals, and transplant centers. Prior dedicated process and policy implementation strategies have been successful in organ donation, and national NRP policies exist.^22,23,26^ As the US NRP experience is still growing, and nearly half of OPOs reported proceeding with NRP without an approved policy, standardized guidelines and policies to reduce operational heterogeneity and improve safety are desperately needed. Our data suggest standardization would decrease the likelihood of allocation challenges, complaints of poor communication, and perhaps even technical failures, as all were significantly associated with increased case volume. Positive steps toward national standardization of NRP technical and ethical standards have been taken by the American Society of Transplant Surgeons (ASTS), and guidelines for the nontechnical and technical aspects of both TA-NRP and A-NRP were presented at the 2024 Developing National NRP Best Practices & Approaches Forum in Denver, Colorado.^27,28,29,30,31^

The variability of NRP experience and associated intercase variability will likely decrease over time. Moreover, the fact that an OPO was primarily prompted to facilitate NRP by a transplant center outside their donor service area, coupled with the fact that 69% of cases were TA-NRP, suggests a relatively small number of heart transplant centers have disproportionately driven the expansion of NRP across the US. To maximize DCD potential, OPOs will need to increase their role as primary drivers of NRP, particularly A-NRP. This would minimize variation for transplant centers traveling outside their donor service area and OPOs hosting a transplant center outside theirs.

Establishing NRP as a standard recovery technique also requires adoption at the donor hospital level, and in the present study one-third of OPOs have at least 1 partner donor hospital that will not allow for NRP for assorted reasons, including ethical and legal concerns. There are multiple ways that OPOs can collaborate with donor hospitals that have concerns with NRP. First, OPOs can share data on the widespread adoption of this technique and data that supports ethical and legal acceptability, increased organ utilization, and improved recipient outcomes.^2,6,7,9,11,13,14,15,16,20,21,32^ Second, OPOs can engage donor hospitals in policy development to allow them to develop protocols that are acceptable within their ethical, religious, and legal frameworks, as has been done with the development of DCD. However, this may lead to a patchwork of confusing policies around NRP, emphasizing the need for fundamental consensus recommendations to guide donor hospital policy development. Third, OPOs can offer the transfer of donors to hospitals that allow NRP so donor hospitals do not have to participate. In the current study, 15% of centers routinely transfer DCD donors, with 3 OPOs citing facilitation of NRP recovery as the sole purpose of transfer. Transfer of the donor to a location specializing in NRP recovery has been advocated.^17^ Suggested benefits include: (1) standardized method of organ reperfusion to ensure safety and reproducibility; (2) reduced subjective variability in organ assessment for transplant suitability; (3) standardized WLST by dedicated intensive care unit physicians and staff who understand the process; and (4) focused expertise in a single location with multiple experienced teams in cardiothoracic surgery, cardiology, critical care, anesthesiology, and abdominal transplant surgery.

Communication around the donor operation and associated logistics is paramount in all organ recoveries. Novel perfusion technology adds a new component to this communication. One of the ethical challenges with NRP is that it can create allocation disputes that are not currently clarified in allocation policy. For example, an abdominal team may want to use NRP for the liver, and the thoracic team might want SRR for the lungs. Dual recovery procedures are possible and have been shown to have excellent liver and thoracic outcomes,^33^ but not all transplant and recovery surgeons are familiar with these procedures. Therefore, OPOs are asked to decide which recovery technique or recipient should take priority. As NRP expands further, our data suggest that these conflicts will be more frequent, emphasizing the need for OPOs to have policies to resolve these conflicts.

### Limitations

This study has limitations. These data represent a prior snapshot in a rapidly evolving field and, thus, do not capture the current state of NRP in the US. Limited experience by some OPOs may have reduced their ability to sufficiently answer survey-specific items. Given the intent and nature of the survey, we did not capture granularity on the specifics of allocation changes or technical failure; these merit further investigation. Additionally, the individuals surveyed may not have been present at or involved in all NRP-related cases, limiting their ability to definitely assess positive and negative implications of NRP.

## Conclusions

This survey study of NRP recovery in the US highlighted the need for national standards to ensure quality and preserve public trust in organ donation. While some degree of variability is inevitable, data suggested most OPOs desire and need standardized guidelines to direct NRP protocol development.

## Notes

Supplement 1. **eTable.** Survey Questions

Supplement 2.
Data Sharing Statement
