# Supplementary material for: Normothermic Regional Perfusion Experience of Organ Procurement Organizations in the US
Source: JAMA Netw Open. 2024 Oct 24;7(10):e2440130. doi: 10.1001/jamanetworkopen.2024.40130 (PMC11581661; doi:10.1001/jamanetworkopen.2024.40130)
Supplement: Supplement 1. — eTable. Survey Questions [file jamanetwopen-e2440130-s001.pdf]

Supplemental Online Content

Sellers MT, Philip JL, Brubaker AL, et al. Normothermic regional perfusion experience of organ procurement organizations in the US. *JAMA Netw Open.* 2024;7(10):e2440130. doi:10.1001/jamanetworkopen.2024.40130

eTable. Survey Questions

This supplemental material has been provided by the authors to give readers additional information about their work.

© 2024 Sellers MT et al. *JAMA Network Open.*

Supplementary Table . Survey questions.

AOPO National Survey on NRP

FINAL (11/6/23)

The AOPO NRP Workgroup put together a comprehensive survey, vetted by several experts in the field, around OPO's experience in NRP. The intent is to gain insights to important information about the NRP landscape and highlight areas for improvement.

We are looking to receive one (1) response per OPO so please ensure this survey is completed by the appropriate person such as the Chief Clinical Officer, Medical Director, etc. Our goal is to have 100% responses from each AOPO OPO member, and the data and information will be shared with everyone.

The survey is easy to complete and will take approximately 10-15 minutes if your OPO participants in NRP. In the event your OPO does not participate in NRP, the skip logic in the survey reduces the completion time to under 5 minutes. A response by **November 17th** is appreciated.

Please tell us about you/your organization.

Please enter:

Full Name. (FIRST AND LAST) ___________________________________

Title: ____

Email: ____

Please select your OPO from the list below.

ALOB	Legacy of Hope	NJTO	NJ Sharing Network
AROR	ARORA	NMOP	New Mexico Donor Services
AZOB	Donor Network of Arizona	NVLV	Nevada Donor Network
CAGS	Sierra Donor Services	NYAP	Center for Donation & Transplant
CAOP	OneLegacy	NYFL	Finger Lakes Donor Recovery Network
CASD	Lifesharing	NYRT	LiveOnNY
CORS	Donor Alliance	NYWN	ConnectLife
FLMP	Life Alliance Organ Recovery Agency	OHLB	Lifebanc
FLUF	LifeQuest Organ Recovery Services	OHLP	Lifeline of Ohio
FLWC	LifeLink of Florida	OHOV	LifeCenter Organ Donor Network
GALL	LifeLink of Georgia	OKOP	LifeShare of Oklahoma
HIOP	Legacy of Life Hawaii	ORUO	Cascade Life Alliance
IAOP	Iowa Donor Network	PADV	Gift of Life Donor Program
ILIP	Gift of Hope Organ & Tissue Donor Network	PATF	Center for Organ Recovery & Education
KYDA	Kentucky Organ Donor Affiliates	PRLL	LifeLink of Puerto Rico
MAOB	New England Donor Services	SCOP	We Are Sharing Hope SC

© 2024 Sellers MT et al. *JAMA Network Open.*

MDPC	Infinite Legacy	TNDS	Tennessee Donor Services
MIOP	Gift of Life Michigan	TNMS	Mid-South Transplant Foundation
MNOP	LifeSource	TXSA	Texas Organ Sharing Alliance
MSOP	Mississippi Organ Recovery Agency	UTOP	DonorConnect
MWOB	Midwest Transplant Network	VATB	LifeNet Health
NCCM	LifeShare Carolinas	WALC	LifeCenter Northwest
NCNC	HonorBridge	WIDN	Versiti Organ and Tissue
NEOR	Live On Nebraska	WIUW	UW Organ and Tissue Donation

1. Which of the following types of NRP recoveries has your OPO participated in within your DSA?

[ ] TA-NRP only → **CONTINUE WITH QUESTION 1A**
[ ] A-NRP only → **SKIP TO QUESTION 1B**
[ ] Both TA-NRP and A-NRP → **ASK QUESTIONS 1A AND 1B**
[ ] Our OPO does not allow NRP → **SKIP TO QUESTION 2**

1a. Please enter the total number of TA-NRP cases your OPO has participated in to-date within your DSA.
Please enter a whole number. __________

1b. Please enter the total number of A-NRP cases your OPO has participated in to-date within your DSA.
Please enter a whole number. __________

[SKIP TO QUESTION 3 AFTER ANSWERING QUESTIONS 1A AND/OR 1B]

2. Why doesn't your OPO allow NRP? **(SELECT ALL THAT APPLY)**

[ ] Concerns surrounding legalities related to NRP
[ ] Concerns surrounding ethics related to NRP
[ ] Due to the American College of Physicians (ACP) statement
[ ] Care-giving team members are resistant
[ ] Concern of negative media attention
[ ] Believe the UDDA should change to better accommodate this approach
[ ] Religious concerns
[ ] Other **(PLEASE SPECIFY)** ______________________

(AFTER RESPONDENT ANSWERS Q.2, SKIP TO QUESTION 24)

© 2024 Sellers MT et al. *JAMA Network Open.*

3. Who has driven your participation in NRP recoveries? **(SELECT ALL THAT APPLY)**
   - [ ] Your OPO
   - [ ] A Transplant Center within your DSA
   - [ ] A Transplant Center outside of your DSA
   - [ ] Other (PLEASE SPECIFY): ___________________________

4. Does your OPO have an approved NRP policy/procedure?
   - [ ] Yes
   - [ ] No, however, we will facilitate both TA-NRP and A-NRP cases in the absence of a policy
   - [ ] No, however, we will facilitate ONLY A-NRP cases in the absence of a policy
   - [ ] We currently have an NRP policy drafted and pending approval

[ASK Q.5 ONLY IF TA-NRP OR A-NRP IS SELECTED IN Q.1; OTHERWISE, SKIP TO QUESTION 24]

This next series of questions will ask about donor hospitals within your DSA that prohibit and/or limit NRP recovery.

5. Do you have hospitals within your DSA that prohibit NRP recoveries?
   - [ ] Yes, Hospitals within our DSA **have prohibited** this type of recovery → **ASK Q.6**
   - [ ] No, Hospitals within our DSA **have NOT prohibited** this type of recovery → **SKIP TO Q.9**

6. How many hospitals in your DSA **prohibit NRP** recoveries? **(PLEASE ENTER A SPECIFIC NUMBER)** ___________

7. What percentage of hospitals in your DSA does this represent? **(PLEASE ENTER A SPECIFIC NUMBER)** __________

© 2024 Sellers MT et al. *JAMA Network Open.*

8. For those hospitals that **<u>prohibit NRP</u>** recoveries, which of the following statements best represents why? **(SELECT ALL THAT APPLY)**

[ ] Concerns surrounding legalities related to NRP
[ ] Concerns surrounding ethics related to NRP
[ ] Due to the American College of Physicians (ACP) statement
[ ] Care-giving team members are resistant
[ ] Concern of negative media attention
[ ] Believe the UDDA should change to better accommodate this approach
[ ] Religious concerns
[ ] Other **(PLEASE SPECIFY)** ________________________

9. Do you have Hospitals within your DSA that **limit you** to **only abdominal NRP recoveries?**

[ ] Yes, Hospitals within our DSA **have limited** to only abdominal NRP recovery
→ ASK Q.10
[ ] No, Hospitals within our DSA **have NOT limited** to only abdominal NRP recovery
→ SKIP TO Q.13

10. How many hospitals in your DSA limit you to only abdominal NRP recoveries? **(PLEASE ENTER A SPECIFIC NUMBER)** ___________

11. What **percentage of hospitals in your DSA** does this represent? **(PLEASE ENTER A SPECIFIC NUMBER)** __________

12. For those hospitals that **limit NRP** recoveries, which of the following statements best represents why? **(SELECT ALL THAT APPLY)**

[ ] Concerns surrounding legalities related to NRP
[ ] Concerns surrounding ethics related to NRP
[ ] Due to the American College of Physicians (ACP) statement
[ ] Care-giving team members are resistant
[ ] Concerns of negative media attention
[ ] Believe the UDDA should change to better accommodate this approach
[ ] Religious concerns
[ ] Other **(PLEASE SPECIFY)** ________________________

© 2024 Sellers MT et al. *JAMA Network Open.*

13. Has your OPO experienced any of the following while coordinating an NRP case? **(SELECT ALL THAT APPLY)**

[ ] Poor communication with the Transplant Center performing NRP
[ ] Challenges with organ allocation and differences of opinions from Transplant Centers regarding approach/perfusion technique
[ ] Limited understanding of the NRP process and related needs (i.e. lab requirements, need for blood, etc.)
[ ] Donor hospital administration had a negative reaction to the NRP process
[ ] Donor hospital critical care personnel had a negative reaction to the NRP process
[ ] Donor hospital O.R. personnel had a negative reaction to the NRP process
[ ] OPO operational staff does not embrace the NRP process
[ ] Donor family has concerns related to the NRP process
[ ] Technical failure during the recovery
[ ] Other **(PLEASE SPECIFY)** ________________________

14. Have your donor hospitals provided you with positive feedback about any experiences with NRP?

[ ] Yes, positive feedback regarding NRP → **CONTINUE WITH Q. 14A**
[ ] No feedback regarding positive NRP experiences→ **SKIP TO QUESTION 15**

14a. What was the positive feedback related to NRP? Please characterize it. **(PLEASE BE SPECIFIC)**

____
____

15. Does your OPO provide specific training to your staff regarding NRP?

[ ] Yes → **CONTINUE WITH QUESTION 16**
[ ] No → **SKIP TO QUESTION 18**

16. Who in your organization do you provide NRP training to? **(SELECT ALL THAT APPLY)**

[ ] Transplant/Donation Coordinators
[ ] Family Approach Personnel
[ ] Hospital Services Personnel
[ ] Perfusion/Recovery Personnel
[ ] Other (SPECIFY)

© 2024 Sellers MT et al. *JAMA Network Open.*

17. What does this training consist of? **(PLEASE BE SPECIFIC)**

____

____

18. Does your OPO specifically authorize/disclose (consent) families for NRP?

[ ] Yes → **CONTINUE WITH QUESTION 19**

[ ] No → **SKIP TO QUESTION 20**

19. At what point in the process do you authorize/disclose (consent) the family for NRP?

[ ] We consent all families for NRP during the organ and tissue consent discussion

[ ] We circle back around to the family after a Transplant Center determines they would like to move forward with NRP

(SKIP TO QUESTION 21 AFTER QUESTION 19)

20. If you **do not** authorize/disclose (consent), do you provide the family with a supplemental form highlighting NRP?

[ ] Yes

[ ] No

[ASK Q.21, IF TA-NRP ONLY OR BOTH, SELECTED IN Q.1. OTHERWISE, SKIP TO QUESTION 24]

21. Does your OPO inform families about any of the following related to the TA-NRP procedure? **(SELECT ONE RESPONSE)**

[ ] We inform the NOK that the head vessels will be occluded. → **ASK Q.22**

[ ] We inform the NOK that the heart will likely restart once on circuit. → **ASK Q.23**

[ ] We inform the NOK that the head vessels will be occluded, and the heart will likely restart once on circuit. → **ASK Qs 22 and 23**

[ ] Neither → **SKIP TO QUESTION 24**

[ASK QUESTIONS 22 and 23 FOR EACH RESPONSE SELECTED IN QUESTION 21. IF NONE IN Q.21, SKIP TO QUESTION 24.]

© 2024 Sellers MT et al. *JAMA Network Open.*

22. When informing the NOK that the head vessels will be occluded, do you document this in the Hospital Electronic Medical Record (EMR) and/or the OPO Electronic Donor Record (EDR)? **(SELECT ALL THAT APPLY)**

[ ] Hospital Electronic Medical Record (EMR)
[ ] OPO Electronic Donor Record (EDR)
[ ] Neither

23. When informing the NOK that the heart will likely restart once on circuit, do you document this in the Hospital Electronic Medical Record (EMR) and/or the OPO Electronic Donor Record (EDR)? **(SELECT ALL THAT APPLY)**

[ ] Hospital Electronic Medical Record (EMR)
[ ] OPO Electronic Donor Record (EDR)
[ ] Neither

[QUESTION 24 ASKED OF ALL REGARDLESS OF RESPONSE IN Q.1]

24. Do you transfer DCD donors to a dedicated hospital or Donor Care Unit?

[ ] Yes → **SKIP TO INSTRUCTION BEFORE QUESTION 25**
[ ] No → **SKIP TO QUESTION 26**

[ASK Q.25 ONLY IF TA-NRP OR A-NRP IS SELECTED IN Q.1; OTHERWISE SKIP TO Q.26]

25. If you transfer DCD donors to a dedicated hospital or DCU, do you perform NRP at this facility?

[ ] Yes → **CONTINUE**
[ ] No → **SKIP TO QUESTION 26**

25a. Do you transfer for the sole purpose of NRP?

[ ] Yes
[ ] No

© 2024 Sellers MT et al. *JAMA Network Open.*

[QUESTION 26 ASKED OF ALL REGARDLESS OF RESPONSE IN Q.1]

The next series of questions will compare and contrast how you operationalize a standard DCD versus a DCD involving NRP. We would like to understand your practice as it relates to the location of withdrawal of support, prepping and draping, and pre-mortem sheath or cannula placement.

26. Does your OPO allow support to be withdrawn in the O.R. for DCDs?

[ ] Yes
[ ] No

[ASK Q.27 ONLY IF TA-NRP OR A-NRP IS SELECTED IN Q.1; OTHERWISE SKIP TO Q.28]

27. Does your OPO allow support to be withdrawn in the O.R. for DCDs involving NRP?

[ ] Yes
[ ] No

[QUESTIONS 28 ASKED OF ALL REGARDLESS OF RESPONSE IN Q.1]

28. Does your OPO allow the donor to be prepped and draped for DCDs **prior to the withdrawal of support**?

[ ] Yes → **SKIP TO QUESTION 30**
[ ] No → **CONTINUE WITH QUESTION 29**

29. Does your OPO allow the donor to be prepped and draped for DCDs **during the five-minute hands-off period**?

[ ] Yes
[ ] No

[ASK Q.30 ONLY IF TA-NRP OR A-NRP IS SELECTED IN Q.1; OTHERWISE SKIP TO Q.32]

30. Does your OPO allow the donor to be prepped and draped for DCDs involving NRP **prior to the withdrawal of support**?

[ ] Yes → **SKIP TO QUESTION 32**
[ ] No → **CONTINUE WITH QUESTION 31**

31. Does your OPO allow the donor to be prepped and draped for DCDs involving NRP **during the five-minute hands-off period**?

[ ] Yes
[ ] No

© 2024 Sellers MT et al. *JAMA Network Open.*

[QUESTION 32 ASKED OF ALL REGARDLESS OF RESPONSE IN Q.1]

32. Does your OPO allow premortem sheath or cannula placement for DCDs?

[ ] Yes→ **CONTINUE WITH QUESTION 32a**

[ ] No→ **SKIP TO QUESTION 33**

32a. Does your OPO **secure consent** for premortem sheath or cannula placement for DCDs?

[ ] Yes
[ ] No

[ASK Q.33 ONLY IF A-NRP IS SELECTED IN Q.1; OTHERWISE SKIP TO INSTRUCTION BEFORE Q.34]

33. Does your OPO allow premortem sheath or cannula placement for **DCDs involving A-NRP**?

[ ] Yes→ **CONTINUE WITH QUESTION 33a**

[ ] No→ **SKIP TO QUESTION 34**

33a. Does your OPO **secure consent** for premortem sheath or cannula placement for **DCDs involving A-NRP**?

[ ] Yes
[ ] No

[ASK Q.34-41 ONLY IF TA-NRP OR A-NRP IS SELECTED IN Q.1; OTHERWISE SKIP TO Q.43]

34. Have you had **organ allocation challenges related to the use of NRP** (i.e., one Transplant Center wants to utilize NRP while another Transplant Center wants to use machine perfusion)?

[ ] Yes → **CONTINUE WITH QUESTION 35**

[ ] No → **SKIP TO QUESTION 37**

35. Thinking about the **organ allocation challenges related to NRP** you have experienced, how would you best characterize them? **(SELECT ONE RESPONSE)**

[ ] These allocation challenges happen routinely and need to be addressed immediately for NRP to be embraced
[ ] While we experience allocation challenges, they occur intermittently and are easily addressed
[ ] We rarely experience any allocation challenges related to NRP
[ ] We believe that NRP is now a standard of care and don't defer to incoming transplant centers if they don't want to do NRP

© 2024 Sellers MT et al. *JAMA Network Open.*

36. When you have **organ allocation challenges related to NRP cases,** how are these challenges typically resolved? **(PLEASE SELECT THE CATEGORY THAT BEST APPLIES)**

[ ] We involve UNOS and allow UNOS to make the final decision
[ ] OPO leadership (AOC and/or Medical Director) makes the final decision
[ ] We allow our local Transplant Surgeon to make the final decision
[ ] We convene a conference call with all the stakeholders to discuss the issue and identify a path forward

Need to clarify q37 - the "...cold perfusion of thoracic organs" applies to OCS hearts too. Lungs would also be a part here but are cold-stored after DPP; also need to reword choice #1 to TA-NRP and liver NMP; no such experience in the US as far as I know with TA-NRP and then OCS for the heart.

37. Has your OPO coordinated NRP cases using any of the following "blended technologies"? **(SELECT ALL THAT APPLY)**

[ ] TA-NRP and OCS/TransMedics
[ ] A-NRP and direct procurement and cold perfusion of thoracic organs
[ ] A-NRP and OCS/TransMedics for thoracic organs
[ ] Other (SPECIFY) ____________________________
[ ] None of the above

38. How do you approach **moving NRP cases forward within the Donor Hospital**? **(SELECT ONE)**

[ ] We request permission to proceed with NRP → **CONTINUE WITH QUESTION 39**
[ ] We do not request permission; we simply notify the hospital → **SKIP TO QUESTION 40**
[ ] We do NOT request permission from and/or notify anyone in the hospital. We believe this is a perfusion technique. → **SKIP TO QUESTION 41**

39. From whom in the Donor Hospital do you **request permission to proceed with NRP**? **(SELECT ALL THAT APPLY)**

[ ] Hospital Leadership
[ ] Chief Medical Officer
[ ] Legal/Risk Management
[ ] Ethics Committee
[ ] Critical Care Team
[ ] O.R. Personnel
[ ] Other **(PLEASE SPECIFY)** ____

© 2024 Sellers MT et al. *JAMA Network Open.*

[SKIP TO QUESTION 41 AFTER ANSWERING Q.39]

40. Who in the Donor Hospital do you **notify about NRP cases**? **(SELECT ALL THAT APPLY)**

[ ] Hospital Leadership
[ ] Chief Medical Officer
[ ] Legal/Risk Management
[ ] Ethics Committee
[ ] Critical Care Team
[ ] O.R. Personnel
[ ] Other **(PLEASE SPECIFY)** ____

41. When an NRP case is planned within your OPO, do you **conduct a pre-arrival huddle (Zoom meeting or conference call) with all the stakeholders** to discuss the plan and NRP procedures?

[ ] Yes→ **CONTINUE WITH QUESTION 41A**
[ ] No → **SKIP TO QUESTION 42**

41a. What are all the considerations reviewed during your **pre-arrival huddle (Zoom or conference call)**? **(SELECT ALL THAT APPLY)**

[ ] Confirm type of NRP, TA-NRP vs. A-NRP
[ ] Review 5-minute hands-off period after death is determined
[ ] Review head vessels will be clamped in TA-NRP cases at the outset of the case
[ ] Review Transplant Team communication expectations while participating in the recovery
[ ] Review the need for a pre-recovery huddle once at the Donor Hospital
[ ] Review the location of the withdrawal of support (O.R. vs. other location)
[ ] Communicate the plan for prepping and draping the patient and the timing approved by the Donor Family
[ ] If more than one team is interested in NRP, determine who will bring the ECMO equipment/personnel
[ ] Review transportation needs (Air/Ground) for Transplant Teams
[ ] Determine each Transplant Team's threshold for functional warm ischemic time (FWIT) and associated cut-off times

© 2024 Sellers MT et al. *JAMA Network Open.*

[ ] Determine if NRP will be pursued if the patient does not die within the specified timeframe for FWIT
[ ] Length of time each team wants to remain on circuit (often heart teams would prefer shorter times compared to liver teams)
[ ] Required labs and who will bring the point of care (POC) device to obtain lab results
[ ] Consider if there are lab requirements of the donor hospital that can't be performed by Transplant Teams with POC device
[ ] Will Transplant Teams bring their own medications vasopressors/inotrope)?
[ ] Will the patient require reintubation, and if so, what is the plan for reintubation?
[ ] Will the heart team need a TEE and if so, what is the plan for facilitating?
[ ] Will the lung team need a Bronch and if so, what is the plan for facilitating?
[ ] Number of PRBCs the Transplant Team needs present in the O.R. from the outset of the case
[ ] Will the patient come off circuit and stay off circuit after heart evaluation? Or will the patient go back on circuit?
[ ] Will Perfusion have additional needs (i.e., oxygen tanks and vacuum lines)?
[ ] Other (SPECIFY) ______________________________________

42. Do you conduct a **<u>pre-recovery huddle with the hospital critical care staff and O.R. personnel</u>** to discuss the logistics and case flow prior to the withdrawal of support?

[ ] Yes
[ ] No

43. How have you handled **<u>hospital education related to NRP</u>**?

[ ] We've decided to educate hospital personnel in real time if an NRP case presents itself
[ ] We've incorporated NRP education into our current professional education offerings.
[ ] We've targeted specific hospitals to provide NRP training
[ ] We currently **<u>do not</u>** provide hospitals with any NRP training

© 2024 Sellers MT et al. *JAMA Network Open.*

44. Have you **developed specific hospital professional education training materials related to NRP**?

[ ] Yes
[ ] No

45. What **NRP information/resources could AOPO provide your OPO** that you would find most helpful? **(PLEASE SELECT ALL THAT APPLY)**

[ ] Workflow checklist and resource guide
[ ] Most effective practices guidelines
[ ] Training materials for OPO staff
[ ] Training material for Donor Hospital Personnel
[ ] Consent documents for NRP
[ ] NRP language samples
[ ] Guide to navigating C-suite engagement
[ ] General education for non-clinical staff/social workers
[ ] Training and/or technical details for your OPO recovery surgeons
[ ] Training and/or technical details for local transplant center surgeon(s)
[ ] Other **(PLEASE SPECIFY)** ____

46. What additional comments or thoughts related to NRP would you like to share that haven't been discussed in the above questions? **(PLEASE BE SPECIFIC)**

____

____

© 2024 Sellers MT et al. *JAMA Network Open.*
