# Supplementary material for: Normothermic Regional Perfusion Experience of Organ Procurement Organizations in the US
Source: JAMA Netw Open. 2024 Oct 24;7(10):e2440130. doi: 10.1001/jamanetworkopen.2024.40130 (PMC11581661; doi:10.1001/jamanetworkopen.2024.40130)
Supplement: Supplement 2. — Data Sharing Statement [file jamanetwopen-e2440130-s002.pdf]

Data Sharing Statement

Sellers. Normothermic Regional Perfusion Experience of Organ Procurement Organizations in the US. *JAMA Netw Open*. Published October 22, 2024.
doi:10.1001/jamanetworkopen.2024.40130

Data
Data available: No

Additional Information
Explanation for why data not available: Aggregate, deidentified data will be made available upon reasonable request to Roxane L. Cauwels or Marty T. Sellers.
